# Trends in Language Assistance for Adults With Non–English Language Preference in California

**DOI:** 10.1001/jamanetworkopen.2025.34741

**Published:** 2025-09-30

**Authors:** Miguel Linares, Jorge A. Rodriguez, Lipika Samal

**Affiliations:** 1 Division of General Internal Medicine and Health Services Research, David Geffen School of Medicine, University of California, Los Angeles; 2 Division of General Internal Medicine, Brigham and Women’s Hospital, Boston, Massachusetts; 3 Harvard Medical School, Boston, Massachusetts

## Abstract

This survey study examines trends in language assistance reported by adults with non–English language preference since the 2016 language access provisions of the Patient Protection and Affordable Care Act.

## Introduction

Language discordance between patients and clinicians contributes to suboptimal health outcomes, yet interpreter use remains inconsistent and is often supplanted by untrained ad hoc interpreters (eg, family members, staff).^1^ The language access provisions of Section 1557 of the Patient Protection and Affordable Care Act, enacted July 2016, built on Title VI language access provisions by explicitly mandating the use of qualified interpreters and translators and prohibiting reliance on untrained individuals for interpretation, except in emergencies.^2^ This analysis examined trends in language assistance reported by adults with non–English language preference and considered whether changes occurred following the 2016 mandate.

## Methods

This survey study used 2011-2023 California Health Interview Survey data that focused on adults (aged ≥18 years) with a non–English-language preference, a typical source of medical care, and a need for someone to help them understand the physician and who answered the question, “Who helped you understand your doctor?” As this analysis used publicly available, deidentified data, the Brigham and Women’s Hospital Institutional Review Board deemed it exempt from review and informed consent. This study followed the STROBE reporting guideline.

Missing data on key study variables (eg, age, sex, race and ethnicity) were handled via complete case analysis. All analyses applied survey weights to account for the survey’s complex sampling design. Percentages of individuals bridging patient-physician communication gaps were plotted by year to display trends over time. Interrupted time series analysis (ITSA) was used to estimate changes in self-reported methods of communication before and after the mandate.

Data were analyzed using Stata, version 17.0 (StataCorp LLC). A 2-sided *P* < .05 was considered significant.

## Results

A weighted sample of 6 298 148 participants across 13 years was analyzed (44.5% aged 18-49 years and 55.6% aged ≥50 years; 59.1% female and 40.9% male; 16.0% self-identified as Asian, 82.7% as Hispanic/Latino, and 1.3% as other [American Indian or Alaska Native, Black, White, or multiracial] race and ethnicity). Of participants needing assistance to understand their physician in the pooled analytic sample, 18.0% interacted with professional interpreters, 53.6% received assistance from medical staff, 17.5% relied on an adult family member or friend, 6.3% used nonmedical office staff, 1.9% reported no assistance, 1.5% indicated other means (eg, other patients), and 1.1% reported a minor child as interpreter.

There were notable trends in the proportion of participants using professional interpreters, rising from 8.3% in 2011 to 34.9% in 2023, while assistance from medical staff declined from 62.0% to 27.8% over the same period (Figure). The ITSA (Table) showed a significant post-2016 trend increase only for nonmedical office staff assistance (annual change estimate, 2.15 percentage points; 95% CI, 0.27-4.03 percentage points; *P* = .03).

**Figure.**
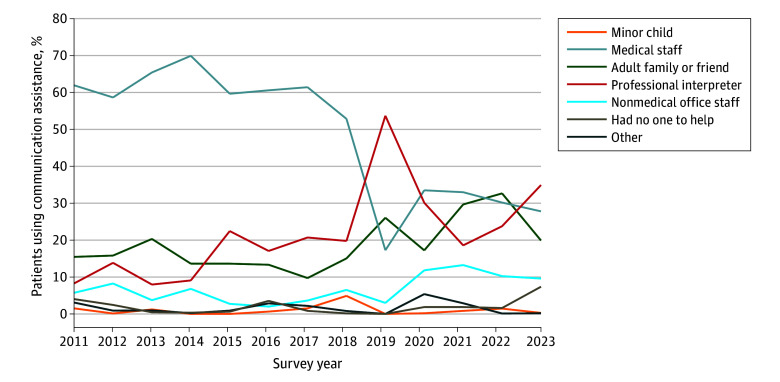
Annual Self-Reported Percentages of Individuals Helping to Bridge Communication Among Patients With Non–English Language Preference, California Health Interview Survey 2011-2023

**Table.** Interrupted Time Series Analysis Estimates of the Annual Percentage of Individuals Helping to Bridge Communication Barriers Before and After the 2016 Mandate of ACA Section 1557, CHIS 2011-2023

Individual who helped the patient understand the physician	Annual change estimate (95% CI), percentage points	*P* value
**Adult family member or friend**		
Trend in and before 2016	−0.69 (−3.70 to 2.33)	.62
Level change after 2016	−1.9 (−15.94 to 12.14)	.77
Trend change after 2016	3.09 (−0.75 to 6.94)	.10
**Nonmedical office staff**		
Trend in and before 2016	−0.91 (−2.39 to 0.55)	.19
Level change after 2016	0.71 (−6.14 to 7.57)	.82
Trend change after 2016	2.15 (0.27 to 4.03)	.03
**Medical staff**		
Trend in and before 2016	0.02 (−5.30 to 5.33)	.99
Level change after 2016	−7.27 (−31.99 to 17.45)	.52
Trend change after 2016	−4.78 (−11.55 to 1.99)	.15
**Professional interpreters**		
Trend in and before 2016	2.02 (−3.72 to 7.77)	.45
Level change after 2016	8.74 (−17.99 to 35.48)	.48
Trend change after 2016	−1.60 (−8.92 to 5.73)	.63
**No assistance**		
Trend in and before 2016	−0.23 (−1.18 to 0.73)	.60
Level change after 2016	−2.78 (−7.22 to 1.66)	.19
Trend change after 2016	1.07 (−0.14 to 2.29)	.08
**Minor child**		
Trend in and before 2016	−0.16 (−0.87 to 0.55)	.62
Level change after 2016	2.51 (−0.79 to 5.80)	.12
Trend change after 2016	−0.19 (−1.09 to 0.71)	.65

Abbreviations: ACA, Patient Protection and Affordable Care Act; CHIS, California Health Interview Survey.

## Discussion

This survey study found that despite a nonsignificant ITSA trend, the steady increase in professional interpreter use over the study period may capture incremental implementation of the Section 1557 mandates and growing staff awareness after COVID-19.^3^ Notably, language access provisions of Section 1557 have undergone rule-making changes, including 2020 rollbacks that weakened definitions, ad hoc restrictions, and tagline (short translated statement about interpreter rights on health documents) requirements.^2^ Notwithstanding, the core mandate to use qualified interpreters and discourage use of untrained individuals first enacted in July 2016 has persisted, justifying a mid-2016 ITSA break. While the ITSA showed no significant difference in the use of adult family members or friends as ad hoc interpreters, these individuals’ persistent involvement raises important concerns given the associated risks of communication errors.^4^ Similarly, frequent use of medical and nonmedical staff underscores the need to certify bilingual staff to minimize interpretation inaccuracies.^5^ The persistent use of children in interpreter roles, while rare, raises ethical concerns.^6^

This analysis is limited by the lack of health care setting details, potential recall and misclassification bias from self-reported data, and regional differences in interpreter availability. Additionally, the 2019 transition of the California Health Interview Survey to the web may have led to a more educated and higher income respondent pool.

## Notes

Supplement.
Data Sharing Statement
