# Supplementary material for: Trends in Language Assistance for Adults With Non–English Language Preference in California
Source: JAMA Netw Open. 2025 Sep 30;8(9):e2534741. doi: 10.1001/jamanetworkopen.2025.34741 (PMC12485635; doi:10.1001/jamanetworkopen.2025.34741)
Supplement: Supplement. — Data Sharing Statement [file jamanetwopen-e2534741-s001.pdf]

Data Sharing Statement

Linares. Trends in Language Assistance for Adults With Non-English Language Preference in California. *JAMA Netw Open*. Published September 30, 2025.
doi:10.1001/jamanetworkopen.2025.34741

Data
Data available: No

Additional Information
Explanation for why data not available: data is publicly available
